# Influence of Alcohol Content and Storage Conditions on the Physicochemical Stability of Spirit Drinks

**DOI:** 10.3390/foods9091264

**Published:** 2020-09-09

**Authors:** Mateusz Różański, Katarzyna Pielech-Przybylska, Maria Balcerek

**Affiliations:** Department of Spirit and Yeast Technology, Institute of Fermentation Technology and Microbiology, Faculty of Biotechnology and Food Sciences, Lodz University of Technology, 90-924 Łódź, Poland; mateusz.rozanski@edu.p.lodz.pl (M.R.); katarzyna.pielech-przybylska@p.lodz.pl (K.P.-P.)

**Keywords:** agricultural distillate, plum distillate, spirit beverages, physicochemical stability, turbidity, volatile compounds

## Abstract

The purpose of this study was to investigate the effects of alcohol by volume (ABV) and storage temperature on changes in the clarity of rye and plum distillates, and their content of volatile compounds. Distillates with initial ABVs of 93.26% *v/v* (rye distillate) and 82.03% *v/v* (plum distillate) were diluted with deionized water to 40, 50, and 70% *v/v*. The samples were stored in darkness at different temperatures (−18 °C, 0 °C, 8 °C, 20 °C) for 8 weeks. The results showed that reducing the alcohol content and storage temperature caused turbidity to increase. The samples prepared from rye distillate were characterized by significantly lower turbidity than those produced from plum distillate. The highest increase in turbidity in comparison to the controls was observed in the samples with 40% *v/v* alcohol content stored at a temperature of −18 °C. Storage of the rye and plum distillates samples at different temperatures resulted in changes to the concentrations of volatile compounds, i.e., lower levels of acetaldehyde and higher alcohols, and increased content of esters. However, the alcohol content and storage temperature had no statistically significant effect on methanol concentration.

## 1. Introduction

Spirit drinks are defined by regulation (European Union, EU) 2019/787 [1] of the European Parliament and of the Council as alcoholic beverages intended for human consumption, possessing particular organoleptic qualities, and containing a minimum alcohol content of 15% *v/v*. Producing spirit drinks typically involves distilling naturally fermented products (potatoes, cereal grains, fruits), maceration or similar processes, and the addition of ingredients, such as flavoring agents or sugars [1].

Grain spirits, including whisky and fruit spirits are made from raw spirits/distillates, which are not purified by rectification but rather aged in wooden barrels, in which their organoleptic qualities are allowed to develop naturally through particular chemical reactions [2].

For instance, Central and Eastern European countries have a long tradition of producing plum distillate-based drinks, known as slivovitz. Due to the different production methods used, especially with regard to fermentation and distillation, plum alcohols can vary greatly from region to region. Sádecká et al. [3] used the technique of synchronous fluorescence spectroscopy (SFS) in combination with principal component analysis (PCA) and linear discriminant analysis (LDA) to differentiate plum spirits, according to their geographical origin. In the case of colorless plum distillates, very good separation of drinks was achieved according to geographical criteria. The results confirmed that the pure plum distillates vary from region to region and showed that SFS can be used to discriminate between plum distillates. Based on the loadings for PCA, volatile phenols and anisols contribute to this discrimination.

The most popular spirit drink in Poland, Russia, and Scandinavia is vodka [4]. Vodka is obtained by diluting ethyl alcohol of agricultural origin with water. The ethyl alcohol is obtained by distilling fermented raw materials, usually distilled/rectified again to obtain a higher alcohol concentration and to selectively reduce volatile by-products of fermentation. The minimum alcohol content in vodka is defined as 37.5% *v/v* [1]. In Poland, the main raw material used in the production of vodka is rye, whereas in Russia it is wheat. Maize, potatoes, molasses, and sugar beet may also be used [5].

Given that the origin of the raw material influences the sensory quality of the rectified spirits that are used to produce vodka, Ziólkowska and Jeleń [6] compared the profiles of the volatile compounds in raw spirits obtained from rye, corn, and potatoes, using two methods based on solid phase microextraction–gas chromatography–mass spectrometry (SPME-GC-MS) and gas chromatography with flame ionization detection (GC-FID). The main groups of volatiles obtained using SPME-GC-MS were fatty acid ethyl esters, whereas using GC-FID fusel alcohols and ethyl acetate dominated. Principal component analysis (PCA) and linear discriminant analysis (LDA) were used for samples differentiation and classification. Potato spirits were found to be relatively easy to differentiate, but it was not possible to fully distinguish the corn samples from the rye samples. The main components of spirit drinks are ethyl alcohol of agricultural origin (rectified spirit) or agricultural distillate (raw spirit) and water. The quality of these components influences the physicochemical stability of the prepared spirit beverages [7].

Apart from ethyl alcohol and water, spirit beverages contain different concentrations of fermentation by-products, depending on the type of spirit used, such as aldehydes, ketones, acids, alcohols, and esters [4,5].

The volatile compounds in a spirit beverage determine its specific aroma and flavor. When spirit beverages with an alcohol content of less than 45% *v/v* are stored under refrigerated conditions, undesirable turbidity, referred to as haze or cloudiness, may appear [8]. This phenomenon is associated with a decrease in the solubility of volatile compounds, such as higher alcohols, and of long chain ethyl esters, such as ethyl dodecanoate, ethyl hexadecanoate, and ethyl-9-hexadecenoate that are present at low concentrations in spirit beverages but may cause flocks or haziness [9,10]. Fatty acid esters behave as surfactants because, as well as a hydrophilic group, they have long hydrophobic carbon chains that prevent them from mixing with water. Under non-mixing conditions, these fatty esters behave as micelles—spherical clumps of lipid molecules [11]. As a rule, haze does not have a significant effect on the smell or taste of spirit drinks. However, it remains a disadvantage, as it contributes to the deterioration of a key trait, namely clarity [12].

To obtain spirit drinks of the highest quality, it is necessary to control the physicochemical composition and organoleptic properties of the ingredients, i.e., spirit and water. Therefore, agricultural distillates are submitted to processes such as rectification and/or filtration [13]. In a process known as ‘cold filtration’, a distillate with an appropriate alcohol content is frozen at a temperature below 0 °C. It can then be filtered to remove turbidity [9,10].

The essential requirement for the water used in spirit drinks that it should meet the requirements for potable water [14]. The quality of water and the water treatment methods used have a significant impact on the physicochemical stability of vodka, and especially on its clarity. High water hardness can cause turbidity in the final product. There are several water treatments available for spirit production, including RO, softening resins, and ion exchange resins [15]. Other processes used are distillation, demineralization, addition of permutate, and softening [1].

Metals, such as aluminum, calcium, cadmium, copper, iron, lead, and potassium can also contribute to the formation of turbidity in spirit drinks. Contact with machines, weighing, bottling, and aging can cause metal ions to migrate into a beverage. Higher concentrations of metals may result in greater turbidity in spirits. Unfortunately, the turbidity may only appear after some time, so it is not possible to state immediately after the production of vodka, whether the product is characterized by high physical and chemical stability [16].

In order to remove impurities causing cloudiness, spirit drinks are filtered through activated carbon, a strongly adsorbent and hydrophobic material [17]. Thanks to these properties, activated carbon is able to trap volatile compounds [12,16]. Moreover, activated carbon catalyzes reactions such as esterification, hydration, and oxidation [12]. Carbon-catalyzed redox reactions also remove undesirable compounds [18]. Vodka may be passed through several layers of activated carbon at an appropriate temperature not less than 0 °C. The contact time with individual layers is also important [19]. Another method uses much lower temperatures, below −45 °C. This helps to prevent the formation of compounds that may spoil the taste of vodka, namely acetals and hemiacetals [20]. It is also possible to use columns filled with active carbon, or carbon may be added directly to the freshly prepared spirit beverage. However, after treatment it is necessary to remove the remaining particles by filtration. The filtration materials should be chosen carefully, to prevent the loss of flavor compounds, which give the spirit its characteristic qualities [12]. Apart from purification, active carbon filtration plays a crucial role in determining the taste of vodka, and vodka producers keep their methods a trade secret [18].

The aim of the present study was to evaluate the effects of the concentration of ethyl alcohol and storage temperature on changes in the clarity of rye and plum distillates (raw spirits), as well as on the qualitative and quantitative composition of volatile compounds in the prepared samples. Determining the most favorable storage conditions for distillates from various raw materials could help to simplify filtration processes.

## 2. Materials and Methods

### 2.1. Raw Materials

The raw materials used were distillates produced on an industrial scale by the Polish companies. Rye distillate (ABV of 93.26% *v/v*) was sourced from the agricultural distillery Zbig-Rol (Prusinowice, Poland). Plum distillate (ABV of 82.03% *v/v*) was provided by the company +H_2_O (Chociszew, Poland). Deionized water was produced using the Simplicity^®^ Ultrapure Water System, 18.2 MΏ·cm resistivity (25 °C) at 0.5 L/min, Merck Millipore (Burlington, MA, USA).

### 2.2. Preparation of Samples

The distillates were diluted with deionized water to ABVs of 40, 50, and 70% *v/v*. Samples of 0.3 L in volume were placed in 0.5 L bottles made from clear soda glass with a glass stopper for an airtight seal. The samples were stored at different temperatures, in darkness, for 8 weeks. After 4 and 8 weeks, the turbidity of the samples was determined, as well as the profile of volatile compounds. The control samples were distillates diluted prior to storage with deionized water. Before storage, control tests were performed, including assessments of both the clarity and of the qualitative and quantitative composition of the volatile compounds. These tests were repeated after 4 weeks of storage and after completion of the test period, i.e., after 8 weeks.

### 2.3. Turbidity Measurement

Turbidity was measured based on the ISO 7027-1:2016 standard “Water quality—Determination of turbidity” [21]. A spectrophotometer was used to determine turbidity in terms of the degree of light transmission through the tested solution (transmittance), at a wavelength of 550 nm. The spectrophotometer was calibrated against demineralized water. A calibration curve was prepared on the basis of the transmittance value for five standard solutions with different turbidities: 0.4, 0.8, 1.2, 1.6, and 2.0 Formazin Turbidity Unit (FTU). The solutions were prepared using 100 FTU formazin solution (Sigma Aldrich, St. Louis, MO, USA) and demineralized water [21].

### 2.4. Chromatographic Analysis

The quantitative composition of selected volatile compounds was tested using an Agilent 7890A gas chromatograph with an Agilent MSD 5975C mass spectrometer (Single Quadrupole), and an HP-5 MS capillary column with dimensions of 30 m × 0.25 mm × 0.25 μm (Agilent Technologies, Santa Clara, CA, USA).

Gas chromatograph (GC) operating parameters were as follows: dispenser temperature—250 °C, sample volume—1 μL, direct injection split—1:40, GC oven temperature program—from 35 °C (6 min) to 100 °C at a rate of 5 °C/min, and then increased to 230 °C at a rate of 10 °C/min (hold time: 2 min), flow rate of the carrier gas (helium) through the column—1 mL/min.

Mass detector (MS) operating parameters were as follows: ion source temperature—230 °C, transfer line temperature—250 °C, ionization energy—70 eV, the mass range during qualitative analysis in the SCAN mode was 20–230 u, dwell time—15 ms. Quantitative analysis was performed in the SIM mode:

1. 1-propanol (monitored ions: 31 *m*/*z*, 59 *m*/*z*, 42 *m*/*z*).
2. Ethyl acetate (monitored ions: 43 *m*/*z*, 61 *m*/*z*, 45 *m*/*z*).
3. 2-methyl-1-propanol (monitored ions: 43 *m*/*z*, 41 *m*/*z*, 42 *m*/*z*).
4. 3-methyl-1-butanol (monitored ions: 55 *m*/*z*, 70 *m*/*z*, 42 *m*/*z*).
5. 2-methyl-1-butanol (monitored ions: 57 *m*/*z*, 56 *m*/*z*, 41 *m*/*z*).
6. Isoamyl acetate (monitored ions: 43 *m*/*z*, 70 *m*/*z*, 55 *m*/*z*).
7. Ethyl hexanoate (monitored ions: 88 *m*/*z*, 43 *m*/*z*, 99 *m*/*z*).
8. Ethyl octanoate (monitored ions: 88 *m*/*z*, 101 *m*/*z*, 43 *m*/*z*).
9. Ethyl decanoate (monitored ions: 88 *m*/*z*, 101 *m*/*z*, 60 *m*/*z*).
10. Ethyl tetradecanoate (monitored ions: 88 *m*/*z*, 101 *m*/*z*, 43 *m*/*z*).
11. Acetaldehyde diethyl acetal (monitored ions: 43 *m*/*z*, 103 *m*/*z*, 73 *m*/*z*).

A 7890A gas chromatograph with a flame ionization detector (FID) was used to determine the content of methanol and acetaldehyde. An HP-INNOWax capillary column with dimensions of 30 m × 0.25 mm × 0.25 µm (Agilent Technologies, Santa Clara, CA, USA) was used to separate the compounds.

Gas chromatograph (GC) operating parameters were as follows: dispenser and detector temperature—250 °C, sample volume—1 μL, direct injection split—1:50, GC oven temperature program—from 40 °C (4 min) to 195 °C at a rate of 10 °C/min (hold time—2 min), flow rate of the carrier gas (helium) through the column—1.5 mL/min.

Quantitative analysis was performed using Agilent MassHunter software (Version B.07.00, Agilent Technologies Inc., Santa Clara, CA, USA) and the external calibration method.

### 2.5. Statistical Analysis

All samples were prepared and analyzed in triplicate. Statistical analyses were performed using Statistica 10 software (TIBCO Software, Palo Alto, CA, USA). The results are expressed as average ± SD. The results were evaluated using analysis of variance (ANOVA) followed by Tukey’s post-hoc test with a significance level of *p* = 0.05 to verify statistical differences.

## 3. Results and Discussion

### 3.1. Turbidity Measurements of the Distillates during Storage

The results of turbidity measurements before storage, and after 4 and 8 weeks of storage, are shown in Table 1.

No turbidity was observed before storage in the case of rye distillate samples with alcohol contents of 40, 50, and 70% *v/v* (control samples). Measurements after 4 weeks of storage showed that turbidity had increased to 0.02 FTU in samples with an ABV of 40% *v/v* stored at temperatures of 8 °C, 0 °C, and −18 °C. In a sample with the same ABV but stored at 20 °C, the turbidity value was 0.01 FTU. After a further 4 weeks, an increase in turbidity was observed in all samples with alcohol contents of 40 and 50% *v/v*. The highest increases in turbidity, to 0.55 and 0.59 FTU, were observed in samples with an ABV of 40% *v/v*, stored at 0 °C, and −18 °C, respectively. These changes in turbidity were statistically significant (*p* < 0.05) compared to the control samples. In the case of rye distillate with an ABV of 70% *v/v*, no turbidity was noticed after 8 weeks of storage, regardless of the temperature.

The turbidity in the control samples prepared from plum distillate with ABV of 40% *v/v* was 0.02 FTU. This was most probably due to the higher (*p* < 0.05) content of volatile compounds in the plum distillate in comparison to the rye distillate. Volatile compounds in the spirit give the final product its specific character. However, when the alcohol content is less than 45% *v/v*, they can cause turbidity, especially during storage [8].

No turbidity was observed in the control samples with alcohol contents of 50 and 70% *v/v*. After 4 weeks of storage, the highest turbidity, ranging from 1.37 FTU (at 20 °C) to 5.41 FTU (at −18 °C) was found in the samples with an ABV of 40% *v/v* (*p* < 0.05). The samples with alcohol contents of 50 and 70% *v/v* stored at 8 °C showed very low turbidity. However, the changes were not statistically significant (*p* > 0.05). After 8 weeks of storage, the turbidity increased in all plum distillate samples, except for two, which had ABVs of 50 and 70% *v/v*, stored at 20 °C. The highest increase in a turbidity in comparison to the controls was observed in the sample with 40% *v/v* alcohol content, stored at a temperature of −18 °C. Turbidity may be associated with a decrease in the solubility of volatile compounds, such as higher alcohols and fatty acid esters (ethyl laurate, ethyl palmitate, ethyl palmitoleate, ethyl myristate), especially at low temperatures [10]. Moreover, the solubility of these compounds decreases as the concentration of ethyl alcohol is reduced [8]. The patterns of changes in clarity observed in the present study are consistent with the results of our previous work [16] and with those reported by other authors [8,10].

### 3.2. Chromatographic Analysis of Distillates during Storage

Changes in the qualitative and quantitative composition of volatile fermentation by-products in the samples of rye and plum distillates with ABVs of 40, 50, and 70% *v/v* were monitored over 8 weeks of storage at different temperatures. The results are shown in Table 2, Table 3, Table 4 and Table 5.

#### 3.2.1. Aldehydes and Alcohols

Acetaldehyde is an intermediate product of ethyl alcohol synthesis in yeast cells during the ethanol fermentation process. It is formed by the decarboxylation of pyruvate, and then reduced to ethyl alcohol. It can also be produced by acetic acid bacteria or by conjugated oxidation of ethyl alcohol and phenolic compounds [22]. The concentration of this compound was significantly lower (*p* < 0.05) in rye distillate (12.52 mg/L alcohol 100% *v/v*) (Table 2) than in the plum distillate (146.67 mg/L alcohol 100% *v/v*) (Table 3). Changes in the concentrations of acetaldehyde in the samples diluted to different ABVs occurred, the intensity of which depended on the concentration of ethyl alcohol and the storage temperature. A higher alcohol concentration in the samples resulted in a greater reduction in acetaldehyde during storage. For rye distillate samples with strengths of 40, 50, and 70% *v/v*, the average decreases in acetaldehyde concentration were about 10%, 12%, and 26%, respectively, compared to the control samples. For analogous samples of the plum distillate, the average decreases were about 19%, 29%, and 57%, respectively. No relationship was found between the reduction of acetaldehyde concentration and storage temperature.

One of the undesirable compounds in spirits is methanol, which is liberated from pectic substances by enzymatic degradation under the influence of a specific pectolytic enzyme, pectin methylesterase, particularly during ripening and fermentation processes. Methanol does not directly affect the flavor of the distillate; however, it is subjected to restrictive controls due to its high toxicity [23,24]. Methanol occurs in all agricultural distillates, except those derived from molasses [25]. In the present study, the rye distillate was found to contain over four times more methanol (300.41 mg/L alcohol 100% *v/v*) (Table 2) than the plum distillate (75.92 mg/L alcohol 100% *v/v*) (Table 3). The higher concentration of methanol in rye distillate compared to the plum distillate may be related to the methods used to prepare the raw materials for fermentation, as well as to the conditions of fermentation and the distillation technology. According to EU Regulation (EC) no. 2019/787 [1], the concentration of methanol in plum distillates should not exceed 12 g/L alcohol 100% *v/v*. In the plum distillate used in our study, the concentration of methanol was significantly below the maximum allowable limit. The concentration of methanol in rye distillates (raw spirits) is not subjected to regulations. The concentrations of methanol in the rye and plum distillates remained at similar levels to the control samples, regardless of the ABV and the temperature. These variables were found to have no statistically significant effect on the methanol concentration (*p* > 0.05).

Agricultural distillates (i.e., raw spirits) contain a relatively large group of compounds known as higher alcohols (fusels), which are present in high concentrations. The presence of these compounds in spirits obtained by ethanol fermentation with the participation of *Saccharomyces cerevisiae* yeast is to be expected, because these microorganisms produce higher alcohols as byproducts of the decarboxylation and deamination of amino acids during the fermentation process. The most representative higher alcohols are 1-propanol, 2-methylpropanol, 3-methylbutanol, and 2-methylbutanol [26].

The samples prepared from rye distillate were characterized by a higher initial content of 1-propanol (Table 2), compared to the samples containing plum distillate (Table 3). After 4 weeks of storage, the content of 1-propanol decreased in all the samples. After the next 4 weeks of storage, a further reduction in the concentration of 1-propanol was observed in the case of the rye distillate samples, to 461.04–514.67 mg/L alcohol 100% *v/v*. The content of 1-propanol increased in two samples of the plum distillate, one with an alcoholic strength of 50% *v/v* (stored at 20 °C) and the other with an ABV of 70% *v/v* (stored at –18 °C) to 127.84 mg/L alcohol 100% *v/v* and 128.52 mg/L alcohol 100% *v/v*, respectively. Moreover, it was noticed that in the samples of rye distillate with an ABV of 70% *v/v*, at all storage temperatures the decrease in propanol content was low (*p* > 0.05). This was in contrast to samples with a 40 or 50% *v/v* alcohol content, in which the changes after storage were greater and statistically significant (*p* < 0.05) (Table 2). A previous study [27] has shown that the propyl alcohol content in distillates obtained from malted and unmalted rye grains may depend on the scale of production scale and processing conditions. The samples prepared on a semi-technical scale were characterized by a lower concentration of propanol (653.89–737.34 mg/L alcohol 100% *v/v*) than those produced industrially (1184.87–2057.90 mg/L alcohol 100% *v/v*) [27]. In another study of plum distillates, the content of 1-propanol was reported as 759.97–1060.50 mg/L alcohol 40% *v/v* [28]. Coldea et al. [29] reported the 1-propanol content in traditional Romanian apple brandy as being 279.7 mg/L alcohol 100% *v/v*.

Isobutyl alcohol (2-methylpropanol) was present in high concentrations in the tested distillates. In the samples of rye spirit, the content of 2-methylpropanol was 699.09 mg/L alcohol 100% *v/v*, whereas in the samples of plum distillate it was 875.14 mg/L alcohol 100% *v/v*. A reduction in the content of 2-methylpropanol was observed in all tested samples, depending on their ABVs and the storage temperatures. In both types of distillates and for all tested ABVs, after 4 weeks of storage, lower temperatures resulted in a greater decrease in the concentration of isobutyl alcohol. However, after the next 4 weeks, no correlation was found between the changes in 2-methylpropanol content and the storage conditions. The changes in the concentration of 2-methylpropanol after 8 weeks of storage were statistically significant (*p* < 0.05), in relation to the results after 4 weeks, except in the case of rye distillates with alcohol contents of 40 and 50% *v/v* stored at 20 °C, and for plum distillate, with an alcohol content of 40% *v/v* stored at 0 °C or 8 °C (Table 2 and Table 3).

Amyl alcohols, i.e., 3-methylbutanol (isoamyl alcohol) and 2-methylbutanol (optically active amyl alcohol) are predominant higher alcohols. They usually constitute at least half of the total amount of this group of volatile compounds [26]. A much higher concentration of amyl alcohols was found in the samples of plum distillate, compared to the samples prepared from rye distillate. The initial content of 2-methylbutanol in the samples of rye distillate was 182.89 mg/L alcohol 100% *v/v*, whereas in the samples of plum distillate it was 672.28 mg/L alcohol 100% *v/v*. The content of 3-methylbutanol was higher, and in the rye distillate samples amounted to 481.46 mg/L alcohol 100% *v/v*, whereas in the plum distillate samples, it was measured at 3098.98 mg/L alcohol 100% *v/v*. Regardless of the storage temperature, the greatest decrease in the concentration of 2-methylbutanol after 8 weeks was noted in the samples with an ABV of 70% *v/v*. In the samples of rye distillate, the concentration of these compounds reduced by approximately 47%, whereas in the samples prepared from plum distillate it fell by about 20% in relation to the control samples. In comparison, the differences in the concentration of 3-methylbutanol were much smaller. The content of isoamyl alcohol in the rye distillate samples decreased by a maximum of about 8%, and that in the plum spirits from about 9% to 17%. In the case of the rye distillate-based samples, there was a greater reduction in the isoamyl alcohol content for lower ABVs. The opposite relationship was observed in the case of plum distillate-based samples. The greatest changes in the isoamyl alcohol content occurred in the trials with an alcohol content of 70% *v/v* and decreased with lower ABVs. The decrease in optically active amyl alcohol concentration was statistically significant (*p* < 0.05) for all samples prepared from both distillates. For isoamyl alcohol, statistically significant changes occurred in all the samples, except for two samples of rye distillate with an alcoholic strength of 50% *v/v*, stored at 20 °C and −18 °C.

#### 3.2.2. Acetals and Esters

When the aldehyde molecule attaches to ethyl alcohol or another alcohol, the aldehyde concentration may be reduced by the formation of acetals [9]. This was observed as an increase in the concentration of acetaldehyde diethyl acetal in the tested samples (Table 4 and Table 5). In the plum distillate sample with an ABV of 70% *v/v*, after the first 4 weeks at all storage temperatures, the concentration of this compound increased more than threefold, from 97.51 mg/L alcohol 100% *v/v* to 337.39−363.20 mg/L alcohol 100% *v/v*. However, after the next 4 weeks, it increased by not more than a further 5% (*p* > 0.05). An increase in the concentration of acetaldehyde diethyl acetal was also observed in the samples of rye distillate, but it was significantly lower compared to the plum distillate. In the samples of rye distillate, the content of this compound before storage was 0.75 mg/L alcohol 100% *v/v*. After 4 weeks, it increased on average to values of around 1 mg/L alcohol 100% *v/v*. After the next 4 weeks, it did not exceed 1.8 mg/L alcohol 100% *v/v*. A relatively large increase in the content of acetaldehyde diethyl acetal was observed only in the sample of rye distillate with 70% *v/v* alcohol content, stored at 20 °C. This finally amounted to 5.22 mg/L alcohol 100% *v/v*. Each change in the concentration of acetaldehyde diethyl acetal was statistically significant (*p* < 0.05). The ABV and storage temperature was found to have no influence on the content of acetaldehyde diethyl acetal in the remaining samples of rye distillate (Table 4).

When analyzing the effect of alcoholic strength and storage temperature on the content of acetaldehyde diethyl acetal in the plum distillate, it was found that the concentration of acetal was higher in the samples with an ABV of 70% *v/v* compared to those with an ABV of 40% *v/v*. Higher storage temperatures resulted in a greater increase in the concentration of acetaldehyde diethyl acetal in the samples with alcohol contents of 40 and 50% *v/v*. However, in the samples with an alcohol content of 70% *v/v*, no strict correlation between storage temperature and changes in acetal content was observed (Table 5).

The decrease in the concentration of higher alcohols in the tested distillates was most probably the result of reactions between alcohols and acids as well as between alcohols and aldehydes, resulting in the formation of esters and acetals, respectively [27]. This hypothesis is supported by the results showing the concentration of esters in the studied distillates (Table 4 and Table 5). A higher initial concentration of all the esters determined in the studied distillates (i.e., ethyl acetate, isoamyl acetate, ethyl caproate, ethyl octanoate, ethyl decanoate, and ethyl myristate) was found in the plum distillate samples (201.10 mg/L alcohol 100% *v/v*) compared to the rye distillate (62.89 mg/L alcohol 100% *v/v*). The largest differences were observed in the case of ethyl acetate, the concentration of which was approximately three times lower (62.12 mg/L alcohol 100% *v/v*) in the rye distillate than in the plum distillate (189.10 mg/L alcohol 100% *v/v*). Such differences result from the high concentration of esters in plum fruits, which may be transferred to the spirit during the distillation process. Literature data [30] confirm that esters are by far the predominant volatiles in plums, accounting for approximately 59% of the total compounds.

In all samples, with all tested ABVs and at all temperatures, the ester content increased over the 8-weeks storage period. In the samples of rye distillate, the greatest increase in the ethyl acetate content was noticed in the samples with an ABV of 70% *v/v*, regardless of the storage temperature. After 8 weeks, it increased by 30% compared to the control sample. In the samples with an alcohol content of 40% *v/v*, the increase in ethyl acetate content was on average 14%, whereas in the samples with an ABV of 50% *v/v* it increased by an average of 24% (*p* < 0.05) (Table 4 and Table 5). The ethyl acetate content in the plum distillate was also correlated with the ABVs of the samples. After 8 weeks of storage, the ethyl acetate content increased by an average of 17% in the samples with 40% *v/v*, by approximately 21% in those with 50% *v/v*, and by 27% in the samples with 70% *v/v*, in comparison to the control samples. The lowest increases in ethyl acetate content were observed in the plum distillate samples with ethyl alcohol concentrations of 40 and 70% *v/v* stored at a temperature of 20 °C. For the former, the concentration of ethyl acetate was 198.67 mg/L alcohol 100% *v/v*, whereas for the latter it was 214.67 mg/L alcohol 100% *v/v* (*p* < 0.05). Exceptionally, the plum distillate sample with an alcoholic strength of 40% *v/v* stored at 20 °C, showed no statistically significant changes (*p* > 0.05) in the concentration of ethyl acetate compared to the control sample.

Isoamyl acetate, which forms as a result of isoamyl alcohol acetyltransferase activity [31], was present in the distillates at much lower concentrations, amounting to 0.52 mg/L alcohol 100% *v/v* in the rye distillate and 1.82 mg/L alcohol 100% *v/v* in the plum distillate. An increase in the alcoholic strength of the samples caused a rise in the concentration of isoamyl acetate, regardless of the storage temperature. In the samples with an alcohol content of 40% *v/v*, the concentration of this compound increased by 16% (rye distillate) and 22% (plum distillate), whereas in the samples with 50% *v/v* it rose by 39% (rye distillate) and 47% (plum distillate). The increase was greatest in the samples with an ABV of 70% *v/v*, reaching 66% (rye distillate) and 74% (plum distillate) relative to the control samples. Generally, the content of isoamyl acetate was higher in the samples prepared from plum distillate, and after 8 weeks, it increased (*p* < 0.05) from 1.82 mg/L alcohol 100% *v/v* to 1.88–3.36 mg/L alcohol 100% *v/v*. In the samples prepared from rye spirit, after 8 weeks of storage the concentration of isoamyl acetate increased from 0.52 mg/L alcohol 100% *v/v* to 0.59–0.91 mg/L alcohol 100% *v/v* (*p* < 0.05).

Higher concentrations of ethyl hexanoate (ethyl caproate) and ethyl octanoate (ethyl caprylate) were also determined in the plum distillate than in the rye distillate (Table 4 and Table 5). The concentrations of these esters in the plum distillate were 0.66 mg/L alcohol 100% *v/v* and 2.37 mg/L alcohol 100% *v/v*, respectively. In the rye distillate, their concentrations were 0.17 and 0.08 mg/L alcohol 100% *v/v*, respectively. In all the tested samples of the plum and rye distillates, an increase in the content of both esters was observed after 8-weeks storage. The samples of rye distillate stored at 20 °C were characterized by the largest increase in the concentration of ethyl caproate, regardless of their alcoholic strength. For example, in the rye distillate with an alcohol content of 70% *v/v* the concentration of ethyl caproate was 0.31 mg/L alcohol 100% *v/v*, whereas in the sample with an ABV of 50% *v/v* it was 0.27 mg/L alcohol 100% *v/v*. In turn, for the samples of rye distillate with 40% *v/v* the concentration of ethyl caproate was 0.22 mg/L alcohol 100% *v/v*. Moreover, in the samples with an ABV of 40% *v/v* stored at 0 °C or −18 °C, the content of ethyl caproate was the same as that determined in the sample stored at 20 °C. All changes in the ethyl caproate concentrations observed during the storage of the plum and rye distillates samples were statistically significant (*p* < 0.05). In the case of plum spirit, the dynamics of the increase in the concentration of ethyl caproate correlated with the ABVs of the tested samples. After 8 weeks of storage, the concentrations of ethyl caproate were within the following ranges: 0.69–1.16 mg/L alcohol 100% *v/v* (40% *v/v*); 1.18–1.40 mg/L alcohol 100% *v/v* (50% *v/v*); 1.46–1.79 mg/L alcohol 100% *v/v* (70% *v/v*). Only in the plum distillate sample with an ABV of 40% *v/v*, stored at −18 °C, did storage not significantly affect (*p* > 0.05) the content of ethyl caproate.

The content of ethyl octanoate (ethyl caprylate) in the rye distillate samples after 8-weeks of storage was noticeably higher in the samples with a lower alcoholic strength. Within the first 4 weeks, changes in the concentration of ethyl octanoate were very small and statistically insignificant (*p* > 0.05). However, over the next 4 weeks, a ten-fold increase (*p* < 0.05) in the concentration of ethyl octanoate was observed. Storage temperature had no significant effect on the concentration of ethyl octanoate. The samples with the same start ABVs stored at all temperature variants ultimately showed similar ethyl octanoate contents. In the samples of rye distillate with an alcoholic strength of 40% *v/v*, the ethyl octanoate contents were in the range of 6.36–6.39 mg/L alcohol 100% *v/v*. An increase in ABV to 50% *v/v* resulted in concentrations of ethyl octanoate in the range of 5.11–5.12 mg/L alcohol 100% *v/v*, whereas for the samples with 70% *v/v* the levels were in the range of 3.66–3.71 mg/L alcohol 100% *v/v*. After 8 weeks of storage, an approximately threefold increase in the concentration of ethyl octanoate was observed in the plum distillate, compared to the controls. It was also noticed that as the storage temperature was lowered, the intensity of ethyl octanoate synthesis decreased, regardless of the ABVs of the tested samples. Only in the samples with an ABV of 50% *v/v* stored at 0 °C and −18 °C were the concentrations of ethyl octanoate similar, at 9.18 mg/L alcohol 100% *v/v* and 8.95 mg/L alcohol 100% *v/v*, respectively.

The presence of ethyl decanoate (caprate) and ethyl myristate was also noted in the plum distillate samples, whereas in the rye distillate these esters were not detected. The decanoate concentration in the plum distillate control sample was 7.09 mg/L alcohol 100% *v/v*, whereas ethyl myristate was present at a much lower concentration of 0.06 mg/L alcohol 100% *v/v*. As with the previously discussed esters, an increase in the content of ethyl decanoate and ethyl myristate was observed (Table 4 and Table 5). Changes in the concentrations of both esters were statistically significant (*p* < 0.05) after storage, compared to the control samples. The concentrations of these compounds increased more sharply when the ABVs of the samples and the storage temperature were higher. As a consequence, the increases in the concentrations of both esters were the largest in the samples stored at 20 °C.

In the case of the plum distillate with an alcoholic strength of 40% *v/v*, the storage temperature was found to have no effect on the changes in the concentration of ethyl caprate. In the samples with ABVs of 50 and 70% *v/v*, an analogous correlation was observed to that for ethyl caprylate. As the storage temperature decreased, the intensity of the increase in the ethyl caprate content decreased. This dependence of changes in the concentration of esters on storage temperature also applied to ethyl tetradecanoate (ethyl myristate).

## 4. Conclusions

In this study, we investigated how ethyl alcohol content and storage conditions influence the clarity and chemical composition of spirit beverages. The richer composition of the plum distillate compared to the rye distillate was reflected by the appearance of higher turbidity during storage of the samples with ABVs of 40, 50, and 70% *v/v*. The highest turbidity was observed in the samples with an ABV of 40% *v/v*, whereas higher alcohol content (50, 70% *v/v*) had a positive effect on the preservation of the clarity of the tested samples. No significant changes in methanol concentration were observed over the 8-week storage period in the rye and plum distillates diluted to ABVs of 40, 50, 70% *v/v*. However, higher ABVs resulted in a more intense decrease in acetaldehyde concentration. No relationship was found between the reduction in acetaldehyde and the storage temperature. Lower concentrations of acetaldehyde were connected with an increase in the concentration of acetaldehyde diethyl acetal. Especially in the samples with ASBVs of 40 and 50% *v/v*, this increase was correlated with storage temperature. A decrease in the concentration of higher alcohols was also noted, as a result of the reaction of alcohols with acids. This resulted in an increase in the concentrations of esters in the tested distillates. The increases in the concentrations of esters were positively correlated to the alcohol concentration of the tested samples. An inverse relationship was observed only for ethyl octanoate in the samples of rye distillate. No strict correlation was found between the storage temperature and the concentrations of esters determined in the distillates.

The results of this study show that the quality of spirit beverages is not only shaped at the production stage, but can also be modified by the storage conditions, as evidenced by the observed changes in the chemical compositions of the tested samples. Our results may be useful for determining the conditions for the preparation of spirit beverages prior to final filtration and bottling. Selection of the appropriate conditions for the storage of distillates could help to avoid the formation of undesirable turbidity, while also maintaining the profile of desirable volatile compounds from the raw plant materials.

## Figures and Tables

**Table 1:** Turbidity changes during storage of samples of rye and plum distillates with different ABVs.

(% *v/v*)	Temp. (°C)	Time (Weeks)	Formazin Turbidity Unit (FTU)	
			Rye Distillate	Plum Distillate
40	Control sample		0.00 ^a^ ± 0.00	0.02 ^a^ ± 0.00
	20	4	0.01 ^b^ ± 0.00	1.37 ^c^ ± 0.00
		8	0.05 ^c^ ± 0.00	1.70 ^d^ ± 0.01
	8	4	0.02 ^b^ ± 0.00	3.22 ^h^ ± 0.02
		8	0.15 ^d^ ± 0.00	4.91 ^i^ ± 0.01
	0	4	0.02 ^b^ ± 0.00	5.58 ^k^ ± 0.02
		8	0.55 ^g^ ± 0.01	6.26 ^l^ ±0.02
	−18	4	0.02 ^b^ ± 0.00	5.41 ^j^ ± 0.02
		8	0.59 ^h^ ± 0.00	6.39 ^m^ ± 0.02
50	Control sample		0.00 ^a^ ± 0.00	0.00 ^a^ ± 0.00
	20	4	0.00 ^a^ ± 0.00	0.00 ^a^ ± 0.00
		8	0.02 ^b^ ± 0.00	0.00 ^a^ ± 0.00
	8	4	0.00 ^a^ ± 0.00	0.02 ^a^ ± 0.00
		8	0.15 ^d^ ± 0.00	2.55 ^e^ ± 0.01
	0	4	0.00 ^a^ ± 0.00	0.00 ^a^ ± 0.00
		8	0.38 ^e^ ± 0.01	2.69 ^f^ ± 0.02
	−18	4	0.00 ^a^ ± 0.00	0.00 ^a^ ± 0.00
		8	0.44 ^f^ ± 0.00	2.78 ^g^ ± 0.02
70	Control sample		0.00 ^a^ ± 0.00	0.00 ^a^ ± 0.00
	20	4	0.00 ^a^ ± 0.00	0.00 ^a^ ± 0.00
		8	0.00 ^a^ ± 0.00	0.00 ^a^ ± 0.00
	8	4	0.00 ^a^ ± 0.00	0.02 ^a^ ± 0.00
		8	0.00 ^a^ ± 0.00	0.09 ^b^ ± 0.00
	0	4	0.00 ^a^ ± 0.00	0.00 ^a^ ± 0.00
		8	0.00 ^a^ ± 0.00	0.11 ^b^ ± 0.00
	−18	4	0.00 ^a^ ± 0.00	0.00 ^a^ ± 0.00
		8	0.00 ^a^ ± 0.00	0.11 ^b^ ± 0.00

Means in a column with a different superscript letters are significantly different (*p* < 0.05) as analyzed by ANOVA and the Tukey’s post-hoc test; n.d.—not detected; ABV—alcohol by volume; Temp.— temperature.

**Table 2:** Changes in aldehydes and alcohols in rye distillate samples during storage at different temperatures.

ABV (% *v/v*)	Temp. (°C)	Time (Weeks)	Compound Concentration (mg/L Alcohol 100% *v/v*)					
			Acetaldehyde	Methanol	1-Propanol	2-Methyl- 1-Propanol	3-Methyl- 1-Butanol	2-Methyl- 1-Butanol
Control sample			12.52 ^k^	300.41 ^abc^	521.00 ^i^	699.09 ^i^	481.46 ^i^	182.89 ^h^
			±0.02	±0.65	±2.22	±0.35	±0.51	±2.00
40	20	4	11.30 ^g^	299.50 ^ab^	507.60 ^gh^	698.76 ^i^	463.27 ^ef^	177.49 ^defgh^
			±0.04	±0.53	±3.11	±1.66	±3.25	±2.57
		8	10.51 ^e^	300.70 ^abc^	483.98 ^de^	697.71 ^i^	456.22 ^cde^	173.64 ^cdefg^
			±0.00	±1.04	±2.48	±3.25	±2.60	±1.42
	8	4	12.43 ^k^	300.29 ^abc^	511.45 ^ghi^	692.65 ^i^	463.51 ^ef^	175.65 ^cdefgh^
			±0.04	±0.87	±1.85	±0.91	±0.92	±0.46
		8	11.86 ^i^	303.20 ^cd^	478.89 ^bcd^	656.91 ^bcde^	457.02 ^cde^	172.94 ^cdefg^
			±0.05	±0.99	±2.69	±4.62	±2.57	±1.48
	0	4	12.43 ^k^	299.65 ^ab^	517.98 ^hi^	676.07 ^gh^	448.98 ^abc^	171.85 ^cdef^
			±0.02	±0.45	±5.20	±4.31	±4.46	±0.82
		8	11.30 ^g^	301.67 ^abc^	491.44 ^ef^	670.55 ^fgh^	445.87 ^ab^	169.33 ^bcd^
			±0.07	±0.61	±2.22	±1.48	±1.97	±2.36
	−18	4	12.43 ^k^	299.46 ^ab^	502.27 ^fg^	674.98 ^gh^	449.94 ^abc^	167.97 ^bc^
			±0.03	±0.58	±6.48	±3.53	±3.82	±2.44
		8	11.30 ^g^	300.61 ^abc^	479.37 ^cd^	665.19 ^defg^	444.02 ^a^	162.06 ^b^
			±0.03	±0.50	±2.43	±3.11	±0.90	±0.72
50	20	4	10.85 ^f^	300.44 ^abc^	485.56 ^de^	698.58 ^i^	474.94 ^ghi^	178.58 ^efgh^
			±0.02	±0.76	±2.22	±1.98	±2.34	±2.66
		8	10.56 ^e^	300.62 ^abc^	471.52 ^abc^	697.64 ^i^	475.39 ^ghi^	173.53 ^cdefg^
			±0.06	±0.28	±2.35	±1.14	±2.84	±1.56
	8	4	12.20 ^j^	302.16 ^bc^	478.90 ^bcd^	666.53 ^efg^	459.29 ^de^	174.12 ^cdefgh^
			±0.03	±0.60	±5.27	±2.79	±4.33	±1.45
		8	11.75 ^hi^	301.24 ^abc^	468.93 ^abc^	650.51 ^bc^	449.94 ^abc^	170.83 ^bcde^
			±0.05	±1.14	±1.01	±3.60	±1.52	±1.71
	0	4	11.75 ^hi^	301.08 ^abc^	466.71 ^a^	649.65 ^bc^	470.43 ^fg^	169.21 ^bcd^
			±0.02	±0.75	±2.35	±3.41	±3.79	±2.63
		8	11.75 ^hi^	303.18 ^cd^	461.04 ^a^	631.98 ^a^	450.52 ^abc^	168.78 ^bcd^
			±0.02	±0.75	±2.21	±3.28	±3.65	±2.47
	−18	4	12.20 ^j^	298.46 ^a^	467.18 ^ab^	654.77 ^bcd^	456.43 ^cde^	170.34 ^bcde^
			±0.06	±0.89	±0.87	±2.12	±2.09	±3.08
		8	9.94 ^c^	299.62 ^ab^	467.15 ^ab^	648.83 ^b^	452.70 ^bcd^	170.34 ^bcde^
			±0.03	±1.40	±1.31	±1.07	±1.60	±2.01
70	20	4	10.33 ^d^	302.31 ^bc^	511.35 ^ghi^	678.64 ^h^	475.30 ^ghi^	180.04 ^fgh^
			±0.04	±0.63	±3.52	±5.98	±2.27	±1.53
		8	10.27 ^d^	302.65 ^bc^	510.14 ^ghi^	671.49 ^gh^	471.15 ^fg^	98.58 ^a^
			±0.05	±0.59	±3.56	±2.21	±1.42	±1.05
	8	4	7.43 ^a^	303.15 ^cd^	520.03 ^i^	671.94 ^gh^	474.85 ^ghi^	181.41 ^gh^
			±0.03	±0.91	±3.70	±1.94	±2.67	±2.32
		8	7.32 ^a^	306.25 ^d^	510.21 ^ghi^	668.09 ^fgh^	469.88 ^fg^	98.22 ^a^
			±0.04	±0.92	±1.20	±1.31	±2.02	±2.30
	0	4	11.62 ^h^	303.03 ^cd^	520.01 ^i^	660.18 ^cdef^	477.48 ^ghi^	175.08 ^cdefgh^
			±0.08	±1.16	±5.01	±1.21	±2.15	±5.61
		8	9.69 ^b^	303.39 ^cd^	514.67 ^hi^	657.07 ^bcde^	472.82 ^gh^	98.98 ^a^
			±0.07	±1.12	±2.13	±5.21	±1.25	±0.86
	−18	4	10.98 ^f^	302.48 ^bc^	518.47 ^hi^	647.04 ^b^	480.70 ^hi^	175.28 ^cdefgh^
			±0.07	±1.00	±3.47	±6.64	±1.38	±3.20
		8	9.69 ^b^	302.74 ^bc^	509.39 ^ghi^	634.49 ^a^	479.77 ^hi^	97.49 ^a^
			±0.04	±1.68	±4.34	±3.76	±1.47	±2.01

Means in a column with a different superscript letters are significantly different (*p* < 0.05) as analyzed by ANOVA and the Tukey’s post-hoc test; n.d.—not detected.

**Table 3:** Changes in aldehydes and alcohols in plum distillate samples during storage at different temperatures.

ABV (% *v/v*)	Temp. (°C)	Time (Weeks)	Compound Concentration (mg/L Alcohol 100% *v/v*)					
			Acetaldehyde	Methanol	1-Propanol	2-Methyl- 1-Propanol	3-Methyl- 1-Butanol	2-Methyl- 1-Butanol
Control sample			146.67 ^l^	75.92 ^ab^	127.46 ^h^	875.14 ^l^	3098.98 ^i^	672.28 ^k^
			±0.45	±0.79	±0.78	±4.53	±15.84	±3.80
40	20	4	128.68 ^ijk^	75.70 ^ab^	124.96 ^gh^	873.64 ^l^	3003.94 ^hi^	668.64 ^jk^
			±0.66	±0.75	±1.20	±2.42	±37.01	±3.15
		8	114.61 ^g^	76.45 ^ab^	119.92 ^ef^	808.41 ^a^	2792.94 ^def^	649.06 ^gh^
			±1.21	±0.94	±1.18	±3.23	±40.31	±3.69
	8	4	129.96 ^jk^	76.28 ^ab^	120.34 ^ef^	871.89 ^l^	2989.13 ^hi^	660.01 ^hijk^
			±1.14	±0.95	±0.67	±4.62	±59.00	±3.60
		8	126.41 ^ij^	75.10 ^ab^	112.65 ^bc^	862.07 ^hijkl^	2836.86 ^efg^	648.92 ^gh^
			±1.60	±0.59	±0.46	±2.84	±36.73	±3.34
	0	4	125.86 ^i^	74.08 ^ab^	120.81 ^ef^	866.96 ^kl^	2982.56 ^hi^	666.21 ^ijk^
			±1.24	±0.34	±0.50	±1.88	±27.56	±2.62
		8	120.93 ^h^	75.09 ^ab^	114.96 ^cd^	863.48 ^ijkl^	2825.16 ^efg^	658.99 ^hijk^
			±0.91	±1.21	±1.23	±2.13	±39.98	±4.04
	−18	4	128.87 ^ijk^	75.76 ^ab^	117.24 ^de^	848.44 ^fgh^	2928.18 ^gh^	645.99 ^fgh^
			±1.09	±0.86	±0.57	±3.02	±24.54	±3.52
		8	115.29 ^g^	75.62 ^ab^	115.07 ^cd^	846.76 ^fg^	2780.53 ^cdef^	631.06 ^f^
			±1.03	±1.10	±1.23	±3.16	±43.35	±3.73
50	20	4	130.95 ^k^	75.57 ^ab^	126.53 ^h^	866.64 ^jkl^	2996.43 ^hi^	659.69 ^hijk^
			±1.44	±0.88	±0.70	±3.60	±34.95	±3.55
		8	118.46 ^gh^	77.30 ^b^	127.84 ^h^	853.20 ^fghijk^	2684.76 ^abcd^	557.09 ^cde^
			±1.18	±1.05	±1.78	±4.33	±46.84	±5.81
	8	4	116.29 ^g^	75.37 ^ab^	108.98 ^ab^	849.06 ^fghi^	2983.47 ^hi^	670.15 ^jk^
			±1.20	±0.60	±0.45	±3.05	±35.97	±4.54
		8	103.26 ^f^	75.85 ^ab^	105.24 ^a^	846.25 ^fg^	2719.84 ^bcde^	571.73 ^e^
			±0.83	±1.02	±1.14	±2.85	±47.08	±3.59
	0	4	107.02 ^f^	75.64 ^ab^	111.15 ^bc^	849.78 ^fghi^	2984.04 ^hi^	655.80 ^hij^
			±2.32	±0.28	±1.64	±2.15	±44.51	±2.96
		8	90.32 ^e^	74.37 ^ab^	110.49 ^b^	847.12 ^fgh^	2696.42 ^bcd^	562.38 ^de^
			±0.47	±0.91	±0.97	±5.20	±25.19	±2.30
	−18	4	115.48 ^g^	76.58 ^ab^	127.07 ^h^	826.07 ^bcd^	2893.13 ^fgh^	635.17 ^fg^
			±0.87	±0.81	±0.80	±4.39	±37.45	±4.72
		8	105.48 ^f^	74.23 ^ab^	126.59 ^h^	821.58 ^abc^	2667.55 ^abcd^	554.44 ^bcd^
			±1.29	±1.18	±0.50	±1.74	±32.67	±3.72
70	20	4	82.03 ^d^	74.40 ^ab^	126.39 ^h^	854.74 ^ghijk^	2996.55 ^hi^	666.53 ^ijk^
			±0.79	±0.48	±1.88	±2.29	±34.50	±3.01
		8	67.35 ^b^	73.35 ^a^	120.67 ^ef^	851.82 ^fghij^	2652.90 ^ab^	541.09 ^b^
			±1.77	±0.84	±1.09	±4.06	±40.42	±4.96
	8	4	71.65 ^c^	75.63 ^ab^	126.08 ^h^	842.15 ^efg^	2996.38 ^hi^	671.17 ^jk^
			±0.98	±0.22	±1.38	±4.22	±31.02	±4.07
		8	57.47 ^a^	75.75 ^ab^	121.77 ^fg^	838.26 ^def^	2642.90 ^ab^	540.92 ^b^
			±0.36	±0.77	±1.43	±1.95	±30.81	±3.82
	0	4	88.69 ^e^	74.80 ^ab^	125.68 ^gh^	830.47 ^cde^	2966.11 ^h^	670.95 ^jk^
			±1.08	±0.84	±0.38	±3.99	±37.32	±4.15
		8	61.10 ^a^	74.52 ^ab^	121.88 ^fg^	828.92 ^cde^	2660.12 ^abc^	541.67 ^bc^
			±1.08	±0.85	±1.09	±2.57	±23.92	±4.88
	−18	4	81.72 ^d^	74.51 ^ab^	126.43 ^h^	811.57 ^ab^	2896.95 ^fgh^	651.13 ^hi^
			±1.18	±0.92	±1.35	±3.58	±25.51	±5.50
		8	65.91 ^b^	75.67 ^ab^	128.52 ^h^	807.03 ^a^	2560.56 ^a^	524.93 ^a^
			±1.60	±0.69	±0.84	±1.36	±39.14	±3.63

Means in a column with a different superscript letters are significantly different (*p* < 0.05) as analyzed by ANOVA and the Tukey’s post-hoc test; n.d.—not detected.

**Table 4:** Changes in acetals and esters in rye distillate samples during storage at different temperatures.

ABV (% *v/v*)	Temp. (°C)	Time (Weeks)	Compound Concentration (mg/L Alcohol 100% *v/v*)						
			Acetaldehyde Diethyl Acetal	Ethyl Acetate	Isoamyl Acetate	Ethyl Hexanoate	Ethyl Octanoate	Ethyl Decanoate	Ethyl Tetradecanoate
Control sample			0.75 ^a^	62.12 ^a^	0.52 ^a^	0.17 ^a^	0.08 ^a^	n.d.	n.d.
			±0.02	±0.78	±0.00	±0.00	±0.00		
40	20	4	1.09 ^bcd^	65.88 ^bc^	0.56 ^bc^	0.22 ^defghi^	0.10 ^a^	n.d.	n.d.
			±0.02	±0.25	±0.00	±0.01	±0.00		
		8	1.68 ^no^	66.32 ^bcd^	0.63 ^de^	0.22 ^cdefgh^	6.39 ^d^	n.d.	n.d.
			±0.05	±0.50	±0.01	±0.02	±0.09		
	8	4	1.08 ^bcd^	70.58 ^fgh^	0.57 ^bc^	0.19 ^abcd^	0.09 ^a^	n.d.	n.d.
			±0.03	±0.93	±0.01	±0.01	±0.00		
		8	1.63 ^lmno^	74.06 ^i^	0.58 ^bc^	0.20 ^abcdefg^	6.37 ^d^	n.d.	n.d.
			±0.05	±0.55	±0.00	±0.00	±0.07		
	0	4	0.98 ^b^	67.32 ^cdef^	0.55 ^ab^	0.20 ^abcdef^	0.09 ^a^	n.d.	n.d.
			±0.05	±0.76	±0.02	±0.01	±0.00		
		8	1.66 ^mno^	69.46 ^defg^	0.59 ^cd^	0.22 ^defghi^	6.36 ^d^	n.d.	n.d.
			±0.05	±1.09	±0.02	±0.01	±0.05		
	−18	4	0.99 ^bc^	67.22 ^cde^	0.58 ^bc^	0.18 ^abc^	0.10 ^a^	n.d.	n.d.
			±0.05	±0.52	±0.01	±0.00	±0.00		
		8	1.47 ^ijkl^	73.52 ^hi^	0.59 ^cd^	0.22 ^bcdefgh^	6.36 ^d^	n.d.	n.d.
			±0.05	±1.03	±0.01	±0.01	±0.04		
50	20	4	1.26 ^efgh^	72.46 ^ghi^	0.80 ^ij^	0.26 ^ij^	0.09 ^a^	n.d.	n.d.
			±0.06	±0.35	±0.02	±0.01	±0.00		
		8	1.78 ^o^	78.14 ^kl^	0.80 ^ij^	0.27 ^j^	5.11 ^c^	n.d.	n.d.
			±0.07	±0.51	±0.00	±0.02	±0.09		
	8	4	1.19 ^def^	70.11 ^efg^	0.73 ^g^	0.18 ^ab^	0.09 ^a^	n.d.	n.d.
			±0.05	±1.24	±0.02	±0.00	±0.00		
		8	1.53 ^jklmn^	79.2^1 klm^	0.75 ^gh^	0.24 ^hij^	5.12 ^c^	n.d.	n.d.
			±0.05	±0.92	±0.01	±0.01	±0.05		
	0	4	1.13 ^bcde^	63.58 ^ab^	0.64 ^ef^	0.19 ^abcde^	0.09 ^a^	n.d.	n.d.
			±0.05	±1.52	±0.01	±0.00	±0.00		
		8	1.57 ^klmn^	72.40 ^ghi^	0.65 ^ef^	0.22 b^cdefgh^	5.11 ^c^	n.d.	n.d.
			±0.04	±0.44	±0.01	±0.01	±0.05		
	−18	4	1.16 ^cdef^	67.30 ^cdef^	0.66 ^ef^	0.18 ^abc^	0.10 ^a^	n.d.	n.d.
			±0.04	±1.12	±0.00	±0.00	±0.00		
		8	1.43 ^hijk^	78.08 ^jk^	0.68 ^f^	0.24 ^ghij^	5.12 ^c^	n.d.	n.d.
			±0.04	±0.79	±0.02	±0.02	±0.02		
70	20	4	2.64 ^p^	74.80 ^ij^	0.87 ^kl^	0.31 ^k^	0.09 ^a^	n.d.	n.d.
			±0.08	±0.71	±0.00	±0.02	±0.00		
		8	5.22 ^q^	81.67 ^m^	0.88 ^lm^	0.31 ^k^	3.71 ^b^	n.d.	n.d.
			±0.04	±0.84	±0.01	±0.00	±0.05		
	8	4	1.40 ^hijk^	72.11 ^ghi^	0.90 ^lm^	0.21 ^bcdefgh^	0.10 ^a^	n.d.	n.d.
			±0.05	±0.65	±0.01	±0.01	±0.00		
		8	1.46 ^ijk^	81.37 ^klm^	0.91 ^m^	0.24 ^ghij^	3.66 ^b^	n.d.	n.d.
			±0.06	±0.80	±0.01	±0.01	±0.06		
	0	4	1.32 ^fghi^	68.67 ^cdef^	0.82 ^ij^	0.21 ^bcdefgh^	0.09 ^a^	n.d.	n.d.
			±0.04	±0.63	±0.00	±0.02	±0.00		
		8	1.49 ^ijklm^	81.40 ^lm^	0.84 ^jk^	0.23 ^efghij^	3.69 ^b^	n.d.	n.d.
			±0.06	±0.76	±0.02	±0.01	±0.06		
	−18	4	1.21 ^defg^	67.23 ^cde^	0.79 ^hi^	0.20 ^abcdef^	0.09 ^a^	n.d.	n.d.
			±0.06	±1.23	±0.00	±0.00	±0.00		
		8	1.37 ^ghij^	80.67 ^klm^	0.81 ^ij^	0.23 ^fghij^	3.68 ^b^	n.d.	n.d.
			±0.04	±0.89	±0.01	±0.01	±0.05		

Means in a column with a different superscript letters are significantly different (*p* < 0.05) as analyzed by ANOVA and the Tukey’s post-hoc test; n.d.—not detected.

**Table 5:** Changes in acetals and esters in plum distillate samples during storage at different temperatures.

ABV (% *v/v*)	Temp. (°C)	Time (Weeks)	Compound Concentration (mg/L Alcohol 100% *v/v*)						
			Acetaldehyde Diethyl Acetal	Ethyl Acetate	Isoamyl Acetate	Ethyl Hexanoate	Ethyl Octanoate	Ethyl Decanoate	Ethyl Tetradecanoate
Control sample			97.51 ^a^	189.10 ^a^	1.82 ^a^	0.66 ^a^	2.37 ^a^	7.09 ^a^ ± 0.02	0.06 ^a^ ± 0.00
			±0.68	±0.97	±0.02	±0.00	±0.01		
40	20	4	102.88 ^ab^	196.26 ^ab^	1.88 ^a^	0.94 ^c^	3.75 ^e^	8.55 ^d^ ± 0.04	0.10 ^def^ ± 0.00
			±1.08	±1.90	±0.02	±0.00	±0.02		
		8	117.52 ^d^	198.67 ^ab^	2.29 ^b^	1.03 ^d^	9.78 ^rs^	9.33 ^f^ ± 0.03	0.14 ^i^ ± 0.00
			±1.32	±1.18	±0.02	±0.01	±0.05		
	8	4	105.42 ^b^	214.57 ^efgh^	1.86 ^a^	0.70 ^a^	3.26 ^c^	7.20 ^a^ ± 0.06	0.09 ^bcd^ ± 0.00
			±1.33	±3.64	±0.02	±0.00	±0.02		
		8	110.95 ^c^	244.65 ^lmn^	2.29 ^b^	1.16 ^fg^	9.58 ^pq^	7.86 ^b^ ± 0.02	0.14 ^hi^ ± 0.01
			±1.61	±3.70	±0.02	±0.01	±0.04		
	0	4	103.85 ^b^	204.76 ^bcdef^	1.90 ^a^	0.88 ^b^	3.44 ^d^	8.17 ^c^ ± 0.06	0.08 ^ab^ ± 0.00
			±1.24	±2.60	±0.02	±0.00	±0.01		
		8	104.90 ^b^	235.25 ^jkl^	2.44 ^cd^	1.14 ^f^	8.73 ^m^	8.77 ^e^ ± 0.06	0.12 ^gh^ ± 0.00
			±1.40	±3.18	±0.02	±0.00	±0.03		
	−18	4	101.50 ^ab^	205.57 ^bcdefg^	1.84 ^a^	0.68 ^a^	2.82 ^b^	8.05 ^bc^ ± 0.06	0.08 ^abc^ ± 0.00
			±1.02	±2.33	±0.00	±0.01	±0.01		
		8	103.80 ^b^	211.64 ^efgh^	1.88 ^a^	0.69 ^a^	8.51 ^l^	8.10 ^c^ ± 0.02	0.12 ^fg^ ± 0.01
			±0.90	±1.54	±0.01	±0.00	±0.06		
50	20	4	180.67 ^f^	216.57 ^hi^	2.50 ^d^	1.08 ^e^	4.85 ^h^	11.95 ^i^ ± 0.00	0.10 ^def^ ± 0.00
			±2.31	±2.76	±0.04	±0.02	±0.04		
		8	188.25 ^g^	226.58 ^ij^	2.62 ^e^	1.25 ^jk^	9.89 ^st^	14.00 ^k^ ± 0.13	0.20 ^kl^ ± 0.00
			±1.53	±2.43	±0.03	±0.01	±0.09		
	8	4	179.13 ^f^	214.30 ^efgh^	2.37 ^bc^	1.02 ^d^	4.80 ^h^	11.44 ^h^ ± 0.04	0.10 ^cde^ ± 0.00
			±1.57	±4.33	±0.01	±0.00	±0.04		
		8	187.52 ^g^	246.64 ^mn^	2.93 ^g^	1.21 ^hi^	9.77 ^rs^	13.19 ^j^ ± 0.04	0.20 ^kl^ ± 0.01
			±1.49	±5.17	±0.02	±0.01	±0.06		
	0	4	175.29 ^f^	201.42 ^bcd^	2.38 ^bc^	1.23 ^ij^	4.61 ^g^	11.26 ^h^ ± 0.03	0.10 ^cde^ ± 0.00
			±1.91	±1.04	±0.03	±0.02	±0.03		
		8	178.96 ^f^	210.93 ^defgh^	2.49 ^d^	1.40 ^m^	8.95 ^n^	11.36 ^h^ ± 0.05	0.19 ^jk^ ± 0.00
			±1.13	±3.25	±0.00	±0.01	±0.06		
	−18	4	169.40 ^e^	200.86 ^bc^	2.32 ^b^	0.91 ^bc^	4.07 ^f^	11.05 ^g^ ± 0.04	0.10 ^def^ ± 0.00
			±1.98	±3.06	±0.03	±0.00	±0.02		
		8	178.07 ^f^	233.53 ^jk^	2.67 ^e^	1.18 ^gh^	9.18 ^o^	11.84 ^i^ ± 0.03	0.18 ^j^ ± 0.00
			±1.98	±3.01	±0.03	±0.02	±0.03		
70	20	4	337.39 ^h^	204.59 ^bcde^	2.99 ^g^	1.37 ^lm^	6.56 ^k^	16.61 ^o^ ± 0.09	0.11 ^efg^ ± 0.00
			±1.96	±2.92	±0.02	±0.00	±0.05		
		8	342.21 ^hi^	214.67 ^fgh^	3.12 ^h^	1.46 ^n^	10.56 ^u^	22.25 ^s^ ± 0.08	0.23 ^m^ ± 0.01
			±2.28	±2.19	±0.04	±0.02	±0.07		
	8	4	363.20 ^k^	219.31 ^hi^	2.94 ^g^	1.34 ^l^	6.64 ^k^	16.35 ^n^ ± 0.01	0.11 ^defg^ ± 0.00
			±1.23	±1.08	±0.01	±0.00	±0.04		
		8	376.92 ^l^	250.72 ^mn^	3.17 ^h^	1.79 ^q^	9.97 ^t^	20.59 ^r^ ± 0.07	0.21 ^l^ ± 0.00
			±1.72	±4.82	±0.03	±0.01	±0.04		
	0	4	360.16 ^k^	215.05 ^gh^	2.84 ^f^	1.28 ^k^	6.18 ^j^	15.74 ^m^ ± 0.05	0.11 ^efg^ ± 0.00
			±1.87	±1.97	±0.04	±0.01	±0.02		
		8	377.47 ^l^	240.80 ^klm^	2.99 ^g^	1.50 ^o^	9.64 ^qr^	20.04 ^q^ ± 0.09	0.21 ^l^ ± 0.01
			±1.88	±3.68	±0.00	±0.02	±0.05		
	−18	4	345.84 ^i^	209.75 ^cdefgh^	2.83 ^f^	1.22 ^hij^	5.38 ^i^	15.21 ^l^ ± 0.02	0.12 ^fg^ ± 0.00
			±0.86	±2.94	±0.03	±0.00	±0.02		
		8	351.96 ^j^	253.68 ^n^	3.36 ^i^	1.55 ^p^	9.44 ^p^	19.04 ^p^ ± 0.08	0.19 ^jkl^ ± 0.00
			±1.47	±2.59	±0.03	±0.01	±0.06		

Means in a column with a different superscript letters are significantly different (*p* < 0.05) as analyzed by ANOVA and the Tukey’s post-hoc test; n.d.—not detected.
